# Lnc_011797 promotes ferroptosis and aggravates white matter lesions

**DOI:** 10.4103/NRR.NRR-D-24-00676

**Published:** 2024-12-16

**Authors:** Xiang Xu, Yu Sun, Xiaoyan Zhu, Shiyin Ma, Jin Wei, Chang He, Jing Chen, Xudong Pan

**Affiliations:** 1 Department of Neurology, Qingdao Central Hospital, University of Health and Rehabilitation Sciences (Qingdao Central Hospital), Qingdao, Shandong Province, China; 2 Department of Neurology, The Affiliated Hospital of Qingdao University, Qingdao, Shandong Province, China; 3 Department of Critical Care Medicine, The Affiliated Hospital of Qingdao University, Qingdao, Shandong Province, China; 4 Department of General Practice, Qingdao Central Hospital, University of Health and Rehabilitation Sciences (Qingdao Central Hospital), Qingdao, Shandong Province, China

**Keywords:** bilateral common carotid artery stenosis, competing endogenous RNA, exosome, ferroptosis, human umbilical vein endothelial cells, long non-coding RNAs, miR-193b-3p, oxygen-glucose deprivation, white matter lesions, WNK1

## Abstract

Recent evidence suggests that ferroptosis plays a crucial role in the occurrence and development of white matter lesions. However, the mechanisms and regulatory pathways involved in ferroptosis within white matter lesions remain unclear. Long non-coding RNAs (lncRNAs) have been shown to influence the occurrence and development of these lesions. We previously identified lnc_011797 as a biomarker of white matter lesions by high-throughput sequencing. To investigate the mechanism by which lnc_011797 regulates white matter lesions, we established subjected human umbilical vein endothelial cells to oxygen-glucose deprivation to simulate conditions associated with white matter lesions. The cells were transfected with lnc_011797 overexpression or knockdown lentiviruses. Our findings indicate that lnc_011797 promoted ferroptosis in these cells, leading to the formation of white matter lesions. Furthermore, lnc_011797 functioned as a competitive endogenous RNA (ceRNA) for miR-193b-3p, thereby regulating the expression of WNK1 and its downstream ferroptosis-related proteins. To validate the role of lnc_011797 *in vivo*, we established a mouse model of white matter lesions through bilateral common carotid artery stenosis. The results from this model confirmed that lnc_011797 regulates ferroptosis via WNK1 and promotes the development of white matter lesions. These findings clarify the mechanism by which lncRNAs regulate white matter lesions, providing a new target for the diagnosis and treatment of white matter lesions.

## Graphical abstract

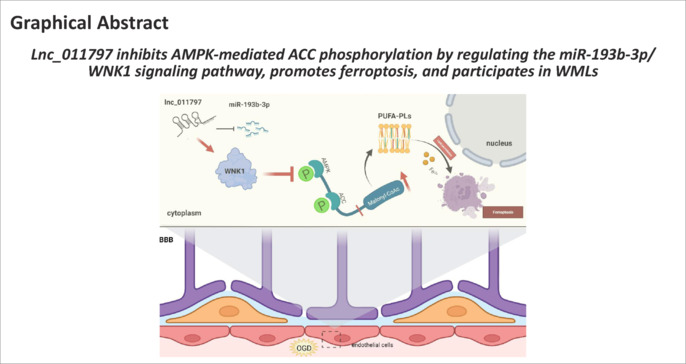

## Introduction

With the aging of the population and the high incidence of cerebrovascular disease risk factors, the incidence of cerebral small vessel disease is increasing annually. White matter lesions (WMLs) are an important pathological feature of cerebral small vessel disease (Kongbunkiat et al., 2017). Clinical manifestations of WMLs include dementia, abnormal gait, and urinary incontinence (Baezner et al., 2008; Kuchel et al., 2009; Hu et al., 2021). However, the pathogenesis of WMLs is still unclear and may be related to destruction of the blood–brain barrier and impaired autoregulation of cerebral blood flow (Lin et al., 2017; Wong et al., 2019). Long-term chronic ischemia and hypoxia can lead to endothelial injury through oxidative stress and other pathways (McCarty, 2015; Forsberg et al., 2018). The major microvascular pathological change seen in regions of white matter subjected to chronic hypoperfusion is endothelial cell injury, which results in myelin loss and gliosis, ultimately inducing white matter injury (Forsberg et al., 2018; Wei et al., 2024). Endothelial cell–oligodendrocyte interactions affect WMLs through multiple pathways (Rajani and Williams, 2017). Given our poor understanding of WMLs, pathogenesis, there are no effective treatment or prevention methods at present. Therefore, clarifying the mechanisms underlying WMLs, pathogenesis is crucial to develop new treatment strategies.

Previous studies have shown that ferroptosis plays a vital role in WMLs, formation and development (Stockwell et al., 2017; Nobuta et al., 2019). Ferroptosis is a form of cell death caused by iron catalytic activity and lipid peroxidation (Stockwell et al., 2017). Ferroptosis has been shown to play an important role in neurological diseases such as stroke and Alzheimer’s disease (Weiland et al., 2019; Tang et al., 2021; Tan et al., 2023). Brain iron levels are elevated with aging and in degenerative disease (Jiang et al., 2017; Reichert et al., 2020). Iron overload can trigger lipid peroxidation in nerve cells, leading to inflammatory activation, myelin degeneration, and a series of related events (Derry et al., 2020; Reichert et al., 2020). Furthermore, ferroptosis can lead to oligodendrocyte loss and demyelination, and ferroptosis inhibitors block oligodendrocyte loss (Jhelum et al., 2020). While it is known that ferroptosis is an important form of cell death in WMLs, the mechanism regulating ferroptosis in WMLs remains unclear. In some cell types, ferroptosis is regulated epigenetically through various pathways, such as long noncoding RNAs (lncRNAs) (Zhang et al., 2022b).

LncRNAs only rarely encode proteins; their primary function is to regulate gene expression in a variety of ways, such as by epigenetic, transcriptional, and posttranscriptional regulation (Kopp and Mendell, 2018; Statello et al., 2021; Gao et al., 2024). Several lncRNAs are known cis- or trans-regulators of ferroptosis (Tang et al., 2021). The lncRNA P53RRA competitively inhibits Ras-GTPase-activating protein-binding protein 1 binding to TP53, which causes cell cycle arrest and ferroptosis (Mao et al., 2018). Linc00336 inhibits ferroptosis in lung cancer by acting as an endogenous sponge for microRNA-6852 (Wang et al., 2019). Approximately 40% of mammalian lncRNAs are expressed in the brain, and lncRNAs play an important role in the central nervous system (Briggs et al., 2015; Tan et al., 2024). LncRNAs can serve as biomarkers for a variety of neurological diseases (Kopp and Mendell, 2018; Zhang et al., 2021). Several studies have shown that lncRNAs play important roles in the WML occurrence and development (Schirmer et al., 2019; Wang et al., 2021). However, it is challenging for lncRNAs in the peripheral circulation to access the central nervous system because they are blocked by the blood–brain barrier and destroyed by RNA-degrading enzymes. It has been demonstrated that exosomal lncRNAs can effectively cross the blood–brain barrier and can be used as biomarkers for neurodegenerative diseases (Kalluri and LeBleu, 2020; Jia et al., 2021; Statello et al., 2021).

Using high-throughput sequencing, we previously showed that a novel exosomal lncRNA, lnc_011797, is a biomarker of WMLs (Xu et al., 2023b). However, the mechanism of action of lnc_011797 in WMLs remains unclear. Bioinformatics analysis suggested that With-no-lysine (K) 1 (WNK1) is a potential target of lnc_011797 (Xu et al., 2023b). Moreover, WNK1 may be associated with ferroptosis. In this study, we established cell and animal models of WMLs to investigate the relationships among lnc_011797, ferroptosis, WNK1, and WMLs formation, to provide new directions for WMLs diagnosis and treatment.

## Methods

### Cell culture

Human umbilical vein endothelial cells (HUVECs; Procell, Wuhan, China, Cat# CL-0675, RRID: cvcl_2959) were cultured in Dulbecco’s modified Eagle medium (Procell) supplemented with 10% fetal bovine serum (Procell) and 1% penicillin-streptomycin (Procell). The cells were cultured in a humid environment at 37°C containing 5% CO_2_.

### Cell transduction

Lnc_011797-overexpressing lentivirus (oe-lnc) and silencing lentivirus (si-lnc) were constructed by Genechem Co. Ltd. (Shanghai, China). A negative control (nc-lnc) were also obtained from Genechem Co. Ltd. To effectively inhibit lnc_011797 expression, we transfected HUVECS simultaneously with lnc_011797 si-lnc #1, si-lnc #2, and si-lnc #3. The details of the lnc_011797 lentiviruses are listed in **Additional Table 1**. Cells (3–5 × 10^5^) were cultured in 6-well plates in preparation for transfection. When the cells reached 40%–60% confluency, 40 µL of infection enhancement solution was added to cells. The HUVECs were divided into the oe-lnc group (transfected with lnc_011797 overexpressing lentiviral vector, multiplicity of infection (MOI) = 50), si-lnc group (transfected with lnc_011797 silencing lentiviral vectors, si-lnc #1 MOI = 30, si-lnc #2 MOI = 30, si-lnc #3 MOI = 30), and nc-lnc group (transfected with lnc_011797 negative control lentiviral vector, MOI = 50). After 72 hours of infection, the cells were cultured in a medium containing 2.0 µg/mL purinomycin for 48 hours to obtain stable cell lines, following the manufacturer’s protocol. The miR-193b-3p mimic, miR-193b-3p inhibitor, and negative controls were synthesized by RiboBio (Guangzhou, China) and transfected into HUVECs using a riboFECT CP transfection kit (RiboBio). The above vectors were transfected into the cells according to the manufacturer’s instructions. In brief, HUVECs are inoculated in 24-well plates and cultured to a density of 30%–50%. The transfection mix was then added to the cells, which were cultured at 37°C for 48 hours (Xu et al., 2023a; Zhi et al., 2023).

**Additional Table 1:** Lentivirus sequence information

Name	Cat#	Sequences (5'-3')
si-lnc#1	101775-11	GCACTTTCCACTACACTTACC
si-lnc#2	101776-11	GCACTCTACACTTAGTTTACT
si-lnc#3	101777-11	GCCTCTACCTATAAATCTTCC
nc-lnc	CON313	TTCTCCGAACGTGTCACGT
oe-lnc	69678-1	TTAAGGAACAAGTGATTATGCTACCTTTGCACGGTTAGGGTACCGCGGCCGTTA AACATATGTCACCGGGCAGGCGGTGCCTCTAATACTGATAATGCTAGAGGTGAT GTTTTTGGTAAACAGGCGGGGTAAGATTTGCCGAGTTCCTTTTACTTTTTTTAAC CTTTCCTTTTGAACTAAGATTCTATCTTGGACAACCAGCTATCACCAGGCTCGAT AGGTTTGTCGCCTCTACCTATAAATCTTCCCACTATTTTGCTACATAGACGGGTG TGCTCTTTTAGCTGTTCTTAGGTAGCTCGTCTGGTACAGGGGTTAGTCCTTGCTG TATTATGCTTGGTTATAATTTTTCATCTTTCCCTTGCGGTACTATATCTATTGCGC CGGTTTACAATTTCTATCGCCTATACTTTATTTGGGTAAATGGTTTGGTTAAGGT TATTTGATAGTAAGGTGGAACGGGTTTAGGGCTAGGTTTGGCTCAGAGTGGTCA AGTTGAGTTGAAATCTCCTAAGTGTAAGTTGGGTGCTTTATGTTAAGCTACACT CTGGTTCGTCCAAGTGCACTTTCCACTACACTTACCATGTTACGACTTGTTTCCT CTATATAAATGCGTAGGGGTTTTAGTTAAATATCCTTTGAAGTATACTTGAGGA GGGTGACGGGCGGTGTGTATGCGCTTCAGGGCCCTGTTCAACTAAGCACTCTAC ACTTAGTTTACTGCTAAATCCACCTTCGACCCTTAAATTTCATAA

### Extraction and identification of human umbilical vein endothelial cells-derived exosomes

Exosomes derived from HUVECs transfected with the lnc_011797 lentiviruses (si-lnc and nc-lnc) were isolated using an exosome isolation kit according to the manufacturer’s instructions (Invitrogen, Carlsbad, CA, USA) (Mao et al., 2019). In brief, HUVEC cell culture medium was collected and centrifuged at 2000 × *g* for 20 minutes at room temperature to remove intact cells and cellular debris. The supernatant was subsequently centrifuged at 10,000 × *g* for 20 minutes to remove any remaining debris. A 0.5 volume of 1× phosphate-buffered saline (PBS) and a 0.05 volume of protease K were added to the supernatant, and the mixtures were then incubated at 37°C for 10 minutes. Next, a 0.2 volume of Exosome Precipitation Reagent was added, and the samples were incubated at 2–8°C for thirty minutes, followed by centrifugation at room temperature at 10,000 × *g* for 5 minutes to precipitate the exosomes. The supernatant was discarded, and the concentration of the exosomes in the pellet was determined by bicinchoninic acid assay (Zhu et al., 2019). Finally, the exosomes were resuspended in PBS and stored at –80°C.

The exosomes were observed and photographed using a transmission electron microscopy (TEM). The size of the exosomes was further confirmed by nanoparticle tracking analysis using a Zeta View PMX 110 (Particle Metrix, Meerbusch, Germany). The expression levels of exosome-specific biomarkers, including two positive markers (CD63 and CD9) and a negative marker (glucose-regulated protein 94, GRP94), were detected via western blotting (Théry et al., 2018).

### *In vitro* oxygen-glucose deprivation insult

A WMLs cell model was established by subjecting cells to oxygen-glucose deprivation (OGD). HUVECs were washed with PBS and then cultured in glucose-free Dulbecco’s modified Eagle medium (Procell) and incubated at 37°C with 95% N_2_ and 5% CO_2_ for 2 hours. The cell culture medium was then changed back to high-glucose Dulbecco’s modified Eagle medium, and the HUVECs were returned to a 95% O_2_ and 5% CO_2_ atmosphere (Choi et al., 2017; Wan et al., 2022; Wang et al., 2024).

### Bilateral common carotid artery stenosis mouse model

All animal experiments performed as part of this study were approved by the Animal Management Committee and Animal Ethics and Welfare Committee of the Affiliated Hospital of Qingdao University on February 18, 2021 (approval No. AHQU-MAL20211126). All experiments were designed and reported according to the Animal Research: Reporting of *In Vivo* Experiments (ARRIVE) guidelines (Percie du Sert et al., 2020). Ten-week-old male C57BL/6J mice (weighing 20–25 g) were obtained from Vital River (Beijing, China, animal license No. SCXK [Jing] 2021-0011). The mice were kept at 18–26°C and 50%–60% humidity with a 12/12-hour light/dark cycle. The animals were divided into two experimental groups: Sham (*n* = 5) and 30-day bilateral common carotid artery stenosis (BCAS, *n* = 15).

The WMLs animal model was constructed by inducing BCAS, as described in previous studies (Shibata et al., 2004; Poh et al., 2021). The mice were anesthetized by isoflurane (1.5%, YUYAN, Shanghai, China) inhalation. The both common carotid arteries were exposed individually, freed from their sheaths, after which microcoils (0.18 mm internal diameter; Sawane Spring Co., Shizuoka, Japan) were wound around the right common carotid artery. After 30 minutes, another microcoil of the same size was twined around the left common carotid arterie. The mice in the BCAS group were injected with 100 μg of exosomes (si-lnc and nc-lnc) or 100 μL of PBS via the tail vein every 5 days after surgery (Tian et al., 2021). Five mice from each group were sacrificed by isoflurane deep anesthesia 30 days after surgery, and the brain tissue was harvested and fixed with 4% paraformaldehyde or 10% neutral buffered formalin for subsequent studies. A flowchart of the animal experiments is shown in **Additional Figure 1**.

### Bioinformatics analysis

Lnc_011797 was identified in our previous study (Xu et al., 2023b). We examined the coding potential of this novel lncRNA using the CPAT database (http://lilab.research.bcm.edu/) (Wang et al., 2013). Recombinant glyceraldehyde-3-phosphate dehydrogenase (GAPDH) was used as the positive control, and metastasis-associated lung adenocarcinoma transcript 1 (MALAT1) was used as the negative control.

### RNA fluorescence *in situ* hybridization

Cy3-labeled lnc_011797 probes were synthesized by RiboBio and hybridized according to the manufacturer’s instructions using a RNA fluorescence in situ hybridization (FISH) kit (RiboBio). Briefly, HUVECs were fixed with 4% paraformaldehyde for 10 minutes and then treated with 0.5% Triton at 4°C for 5 minutes. The cells were then hybridized with lnc_011797 probes at 37°C overnight. Fluorescence images were captured by confocal microscopy (Leica, Weztlar, Germany). RNA FISH was used to evaluate lnc_011797 distribution in HUVECs (Liao et al., 2023b; Zhang et al., 2023).

### Real-time polymerase chain reaction

Total RNA was extracted from HUVECs using Trizol reagent (Qiagen, Frankfurt, Germany). lncRNAs, microRNAs (miRNAs), and mRNAs were reverse transcribed into complementary DNA using a GoldenStar RT6 cDNA Synthesis Kit ver. 2 (TSINGKE, Beijing, China) followed by a Mir-XmiRNA First-Strand Synthesis Kit (Takara, Osaka, Japan). Real-time polymerase chain reaction (RT-PCR) was performed using TB Green® Premix Ex Taq^TM^ II (Takara), and gene expression levels were detected using a LightCycler 480 II. The details of the cycling conditions are listed in **Additional Tables 2** and **3**. The final extension was 72°C for 5 minutes. The specific primers used are shown in **Table 1**. The experiment was carried out in triplicate. The internal controls were ACTB and U6. Relative expression was calculated using the 2^–ΔΔCt^ method (Ding et al., 2024), where ΔCt = Ct_target_ – Ct_reference_, ΔΔCt = ΔCt_treatment_ – ΔCt_control_.

**Additional Table 2:** The PCR condition of miRNA

	Temperature (°C)	Time (s)	Cycle
Predenaturation	95	10	1 cycle
Denaturation	95	5	40 cycles
	60	20	
Melting curves	95	60	
	55	30	1 cycle
	95	30	

miRNA: MicroRNA; PCR: polymerase chain reaction.

**Additional Table 3:** The PCR condition of lncRNA and mRNA

	Temperature (°C)	Time (s)	Cycle
Predenaturation	95	30	1 cycle
Denaturation	95	5	
	55	30	40 cycles
	72	30	
Melting curves	95	1	
	65	60	1 cycle
	95	Continuous	
Cooling	40	1	1 cycle

lncRNA: Long non-coding RNA; PCR: polymerase chain reaction.

**Table 1:** Primer sequences in the study

Gene	Primer sequence (5'–3')
lnc_011797	F: AGG TGG AAC GGG TTT AGG GCT AG
	R: CAC TTG GAC GAA CCA GAG TGT AGC
WNK1	F: CGA GTG AGC AGC CAA CAG ACA G
	R: TGT GCT TGG ACA GTA GAA GGT ATA TGC
miR-193b-3p	F: AGA TCG ATT CGC CCT GAA ACT
ACTB	F: TGC TGT CAC CTT CAC CGT TCC A
	R: GCG GAC TAT GAC TTA GTT GCG TTA CA
U6	F: CTC GCT TCG GCA GCA CA
	R: AAC GCT TCA CGA ATT TGC GT

F: Forward; R: reverse.

### Western blot assay

The exosomes and HUVECs were washed with PBS. Total protein was extracted by incubating with radio immunoprecipitation assay buffer containing phenylmethanesulfonyl fluoride (MedChemExpress, Princeton, NJ, USA) on ice for 30 minutes. The proteins were separated by 12% sodium dodecyl sulfate polyacrylamide gel electrophoresis and transferred onto polyvinylidene fluoride membranes (Millipore Co., Boston, MA, USA). The membranes were incubated with primary antibody at 4°C overnight and then with horseradish peroxidase-conjugated goat anti-rabbit IgG (1:10,000, Abcam, Cambridge, UK, Cat# ab6721, RRID: AB_955447) at 37°C for 1 hour (Cordonnier et al., 2020). The membrane was visualized by chemiluminescence detection (UVITEC, Cambridge, UK). The primary antibodies used included antibodies against β-actin (mouse, 1:1000, Abcam, Cat# ab8226, RRID: AB_306371), WNK1 (rabbit, 1:1000, Abcam, Cat# ab174854, RRID: AB_3492057), acetyl-CoA carboxylase (ACC; rabbit, 1:1000, Abcam, Cat# ab109368, RRID: AB_10864809), phosphor-ACC (p-ACC; phospho S79, rabbit, 1:1000, Abcam, Cat# ab68191, RRID: AB_11156104), AMP-activated protein kinase (AMPK; rabbit, 1:1000, Abcam, Cat# ab32047, RRID: AB_722764), phosphor-AMPK (p-AMPK; phospho T183, rabbit, 1:1000, Abcam, Cat# ab133448, RRID: AB_2923300), CD9 (rabbit, 1:1000, Abcam, Cat# ab236630, RRID: AB_2922400), CD63 (rabbit, 1:1000, Abcam, Cat# ab134045, RRID: AB_2800495), and GRP94 (rabbit, 1:1000, Abcam, Cat# ab238126). ImageJ software (ImageJ bundled with 64-bit Java version 1.8.0_172; National Institutes of Health, Bethesda, MD, USA) (Schneider et al., 2012) was used to calculate the optical density of the bands. Three replicates were included for each group.

### Cell counting kit-8 assay

HUVECs were inoculated into 96-well plates, and 10 µL of cell counting kit-8 (CCK-8) solution (MedChemExpress) was added to each well. After the cells were incubated at 37°C for 2 hours, the absorbance at 450 nm was measured with a microplate reader (Molecular Devices, San Jose, CA, USA) (Mao et al., 2019). Three replicates were included for each group.

### Lipid reactive oxygen species assay

HUVECs were cultured in 6-well plates, and DCFH-Da (Elabscience) was added to each well. The plates were incubated for 30 minutes at 37°C, after which a microplate reader (excitation wavelength 485 nm, emission wavelength 525 nm; Molecular Devices) was used to measure the fluorescence intensity to quantify the reactive oxygen species (ROS). The fluorescence images were captured by a fluorescence microscope (Olympus, Hamburg, Germany), and the exposure time was 380 ms.

### Iron assay

Cellular iron content was determined using an iron assay kit (Elabscience) according to the manufacturer’s instructions. Briefly, 5 × 10^6^ HUVECs were resuspended in PBS and then crushed with an ultrasonic cell crusher (SONICE, New York, NY, USA) on ice. The iron chromogenic agent was added to the supernatant, and the mixture was incubated in a boiling water bath for 5 minutes, followed by centrifugation at 3000 × *g* for 10 minutes. Two hundred microliters of supernatant were added to each well of a 96-well plate, and the optical density (OD) was measured at 520 nm (Qiang et al., 2020). The protein concentration of the supernatants was determined using a bicinchoninic acid (BCA) protein assay (Elabscience). The Fe^2+^ content was calculated as follows: Fe^2+^ (mg/gprot) = (ΔA_520_ – *b*) /*a* × *f*/*Cpr*, where ΔA_520_ is sample OD value – blank OD value, *b* is standard curve intercept, a is standard curve slope, *f* is dilution ratio of sample, and *Cpr* is sample protein concentration (gprot/L).

### Transmission electron microscopy

HUVECs were seeded into T25 culture flasks and subjected to OGD for 2 hours. The cells and exosomes were then fixed with 2.5% glutaraldehyde overnight at 4°C. Images were obtained using an 8JEM-1200EX transmission electron microscope (JEOL, Tokyo, Japan) (Zheng et al., 2023). Mitochondrial length was measured using ImageJ software (Miao et al., 2023).

### Dual-luciferase reporter assay

According to the RNAhybrid database (https://bibiserv.cebitec.uni-bielefeld.de/rnahybrid), miR-193b-3p is predicted to bind to lnc_011797. Using the TargetScan online database (http://www.targetscan.org/vert_72/) (Agarwal et al., 2015), we also predicted that miR-193b-3p could bind to WNK1. Plasmids were constructed using a luciferase reporter vector (GV272) encoding wild-type WNK1 or lnc011797 and WNK1 or lnc011797 sequences mutated at the predicted binding sites (mut-lnc_011797, wt-lnc_011797, mut-WNK1, and wt-WNK1) (Genechem Co. Ltd.). The reporter vectors were cotransfected into HUVECs with the miR-193b-3p mimic or an miR-193b-3p negative control using Lipofectamine 2000 transfection reagent (Thermo Scientific, Waltham, MA, USA). Next, a dual-luciferase reporter assay system (Promega, Madison, WI, USA) was used to measure luciferase activity. Briefly, the cells were lysed with 100 μL of lysis buffer at room temperature for 15 minutes. Then, 20 μL of lysis solution was added to the all-white culture plate, and Luciferase Assay Reagent II and Stop&Glo Regen were added to detect firefly luciferase activity and Renilla luciferase activity, respectively. The relative luciferase activity was calculated as the ratio of firefly luciferase intensity to Renilla luciferase intensity (Mao et al., 2019). Triplicate wells were set up for each group.

### Luxol-fast blue staining

Axonal fiber density was detected by luxol-fast blue (LFB) staining (Solarbio, Beijing, China) to determine WML severity. Briefly, paraffin sections of mouse brain tissues were dehydrated in 95% ethanol and then submerged in LFB staining solution stained at room temperature for 20 hours. Excess stain was removed by treatment with 95% ethanol, followed by washing with deionized water. The sections were then soaked in LFB differentiation solution was for 15 seconds. Gray and white matter differentiation was initiated by treatment with 0.05% aqueous lithium carbonate (Abcam) for 20 seconds, followed by treatment with 70% ethanol until the nuclei were decolorized. Then, the sections were immersed in eosin stain (Solarbio) for 1 minute and washed in deionized water. Next, the sections were dehydrated in an ethanol gradient (70%–100%), cleared with xylene, and mounted with a mounting agent. Bright field images were taken at 4× and 60× magnification using an Olympus upright fluorescence microscope (BX53) (Poh et al., 2021). WML severity was classified as normal (grade 0), disordered arrangement of nerve fibers (grade 1), obvious vacuolation (grade 2), or loss of myelinated fibers (grade 3) (Toyama et al., 2018; Alfieri et al., 2022).

### Hematoxylin and eosin staining

Mouse brains were sectioned and stained with hematoxylin and eosin (Solarbio) for observation under an optical microscope (Olympus).

### Immunohistochemistry

Glial fibrillary acidic protein (GFAP) and WNK1 expression in the mouse brain was detected by immunohistochemistry (Solé-Guardia et al., 2023). GFAP expression indicates astrocyte activation, and GFAP expression levels are significantly elevated in WMLs (Suzuki et al., 2021). Immunohistochemical experiments were performed using a BenchMark ULTRA automatic immunohistochemical apparatus (Roche, Basel, Switzerland). The tissue sections were imaged using an optical microscope (Shibata et al., 2004; Poh et al., 2021; Zhang et al., 2022b). Quantification of immunohistochemical staining was performed using ImageJ software. The antibodies used included: anti-GFAP (rabbit, 1:200, Huabio, Hangzhou, China, Cat# R1308-10, RRID: AB_3073229), anti-WNK1 (rabbit, 1:200, Huabio, Cat# JG36-91, RRID: AB_3070839), and goat anti-rabbit IgG H&L (1:200, Abcam, Cat# ab6721, RRID: AB_2722623).

### Neurobehavioral score

Neurobehavioral changes in mice were mainly evaluated by neurobehavioral score (Qi et al., 2019; Xiao et al., 2024). This experiment was performed one month after BCAS, and each evaluation lasted for 2 hours. Auricle reflex, corneal reflex, righting reflex, tail flexion, and flight response reflex were observed. A normal conditioned reflex score was 2 points, a conditioned reflex score (slower than normal rats) was 1 point, and an unconditioned reflex score was 0 points (Song et al., 2022).

### Statistical analysis

The data were analyzed using GraphPad Prism 8.0 software (GraphPad Software, San Diego, CA, USA, www.graphpad.com). Quantitative variables are expressed as the mean ± standard deviation (SD). Comparisons between the two groups were performed by independent samples *t*-test was used to compare nonnormally distributed data between two groups. Three or more groups were compared using one-way analysis of variance followed by the least significant difference *post hoc* tests. *P* < 0.05 was considered to indicate statistical significance.

## Results

### Lnc_011797 expression is increased in a WML cell model

**Figure 1:**
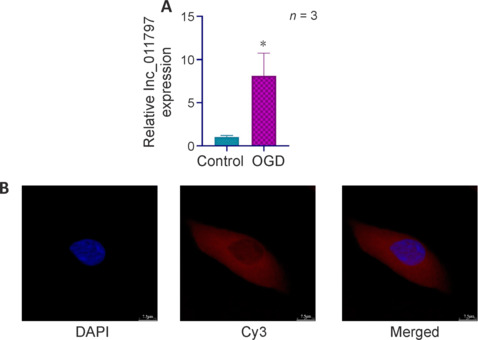
Lnc_011797 is upregulated in cells subjected to OGD. (A) RT-PCR results showing that lnc_011797 expression was upregulated in the OGD group. The data were normalized to the control group. Data are expressed as mean ± SD (*n* = 3). *P < 0.05 (independent samples *t*-test). (B) RNA FISH confirmed that lnc_011797 (Cy3, red) was mainly distributed in the cytoplasm of HUVECs. Scale bars: 7.5 μm. DAPI: 4′,6-Diamidino-2-phenylindole; FISH: fluorescence in situ hybridization; HUVECs: human umbilical vein endothelial cells; OGD: oxygen-glucose deprivation; RT-PCR: real-time polymerase chain reaction.

In our previous study (Xu et al., 2023b), through RNA sequencing, we found that lnc_011797 is a biomarker of WMLs whose expression level is related to disease severity. To verify that lnc_011797 is involved in WMLs, we subjected HUVECs to OGD to create an *in vitro* model of WMLs (Choi et al., 2017) and measured lnc_011797 expression by RT-PCR. Compared with the control group, lnc_011797 expression in the OGD group was significantly greater (*P* = 0.04; **Figure 1A**). This finding is consistent with our sequencing results.

Next, RNA FISH was used to investigate the subcellular localization of lnc_011797. We found that lnc_011797 localized mainly to the cytoplasm (**Figure 1B**). CPAT database analysis indicated that lnc_011797 has no coding potential (**Table 2**).

**Table 2:** The ability of coding

Name	Coding probability	Coding label
lnc_011797	0.0006077	No
GAPDH	0.9944163	Yes
MALAT1	0.0141953	No

GAPDH: Glyceraldehyde-3-phosphate dehydrogenase; MALAT1: metastasis associated in lung adenocarcinoma transcript 1.

### Silencing lnc_011797 reverses ferroptosis in a cell model of WMLs

**Figure 2:**
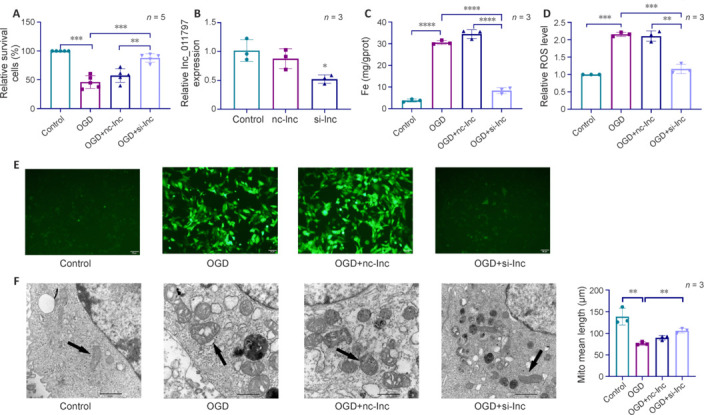
OGD induces ferroptosis in HUVECs. (A) CCK-8 assay showing that OGD decreases HUVEC viability. Cell viability was restored in the si-lnc group. (B) Lnc_011797 si-lnc effectively inhibited lnc-011797 expression. The data were normalized to the control group. (C) The intracellular iron concentration in the OGD group was significantly higher than that in the control group and the OGD + si-lnc group. (D, E) A microplate reader (D) and fluorescence microscope (E) were used to show that the relative ROS concentration (green fluorescence) increased after OGD and decreased after lnc_011797 knockdown. The data were normalized to the control group. Scale bars: 50 μm. Data are expressed as mean ± SD. **P < 0.01, ***P < 0.001, ****P < 0.0001 (one-way analysis of variance followed by the least significant difference *post hoc* tests). (F) Electron microscopy images of mitochondria. The mitochondrial morphology was normal in the control group. Mitochondrial cristae were decreased, membrane density was increased, and average mitochondrial length was decreased in the OGD and nc-lnc groups compared with the control group. The mitochondrial damage was alleviated in the si-lnc group. Scale bars: 1 μm. CCK-8: Cell counting Kit-8; HUVECs: human umbilical vein endothelial cell; OGD: oxygen-glucose deprivation; ROS: reactive oxygen species.

To further investigate the role of lnc_011797 in ferroptosis, we transfected cells with lnc_011797-silenced lentivirus (si-lnc). lnc_011797 expression was successfully reduced in the si-lnc transfected cells compared with the control and nc-lnc groups (**Figure 2A**). Previous studies have shown that OGD can lead to ferroptosis (Yuan et al., 2021; Liao et al., 2023a); therefore, we next tested cell viability using a CCK-8 kit and found that OGD significantly decreased cell viability (*P* = 0.0004; **Figure 2B**). Moreover, silencing lnc_011797 improved the viability of cells subjected to OGD (**Figure 2B**). Then, we used TEM, intracellular iron concentration detection, and ROS level detection to determine whether ferroptosis occurred in cells subjected to OGD. The intracellular iron concentrations and ROS levels in the OGD group were greater than those in the control group (**Figure 2C–E**). TEM showed that the mitochondrial crest decreased or disappeared, the mitochondrial membrane density increased, and the mean mitochondrial length were decreased in the OGD group compared with the control group (**Figure 2F**). These findings indicate that OGD induces ferroptosis and decreases cell viability. However, the intracellular concentration of iron in the OGD + si-lnc group was lower than that in the OGD group (**Figure 2C**). Furthermore, lnc_011797 knockdown reversed the increase in ROS levels caused by OGD (**Figure 2D** and **E**). When lnc_011797 expression was knocked down, OGD-induced mitochondrial lesions were milder (**Figure 2F**). Thus, decreased lnc_011797 expression was associated with ferroptosis inhibition, suggesting that lnc_011797 regulates ferroptosis.

### Lnc_011797 accelerates white matter lesion development by regulating WNK1

**Figure 3:**
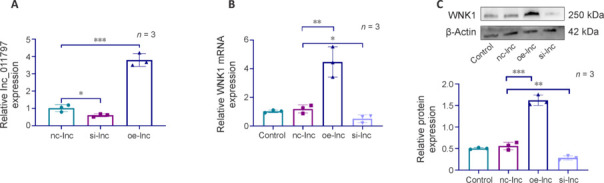
Lnc_011797 regulates WNK1 expression in HUVECs. (A) RT-PCR analysis of lnc_011797 expression in HUVECs. (B) WNK1 mRNA expression after transfection with specific lentiviruses was analyzed via RT-PCR. The data were normalized to the control group. (C) Western blot analysis was used to assess WNK1 expression after transfection with specific lentiviruses. Data are expressed as mean ± SD (*n* = 3). *P < 0.05, **P < 0.01, ***P < 0.001 (one-way analysis of variance followed by the least significant difference *post hoc* tests). HUVECs: Human umbilical vein endothelial cells; RT-PCR: real-time polymerase chain reaction; WNK1: with-no-lysine (K) 1.

We previously reported that lnc_011797 may play an important role in WMLs by targeting WNK1 (Xu et al., 2023b). To study the mechanism by which lnc_011797 promotes WMLs development, we generated stable cell lines in which lnc_011797 was overexpressed or knocked down by lentivirus and verified the lnc_011797 expression level in the different groups by RT-PCR (**Figure 3A**). To determine whether WNK1 was regulated by lnc_011797, we detected WNK1 expression in lentivirus-transfected cells by RT-PCR and western blotting. As expected, WNK1 mRNA and protein expression levels were markedly increased by lnc_011797 overexpression and decreased by lnc_011797 silencing (**Figure 3B** and **C**). Taken together, these findings confirm that lnc_011797 regulates WNK1 expression.

### Lnc_011797 regulates WNK1 expression by acting as a sponge for miR-193b-3p

**Figure 4:**
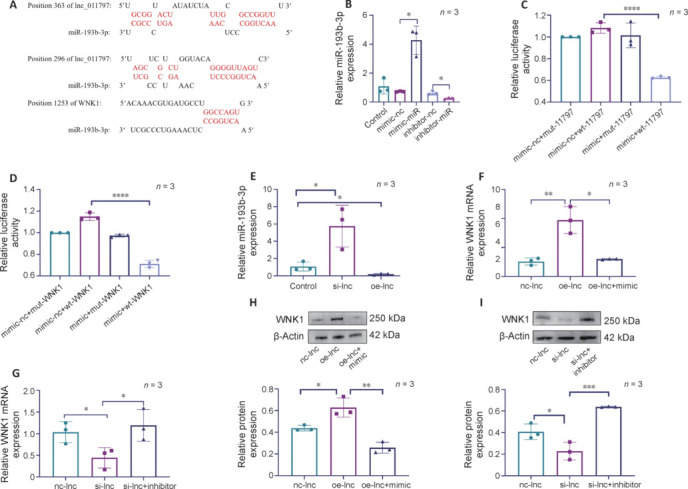
Lnc_011797 can bind to miR-193b-3p to regulate WNK1 expression. (A) Predicted binding sites between miR-193b-3p and lnc_011797 or WNK1. Lnc_011797 contains two predicted miR-193b-3p binding sites (shown in red). (B) RT-PCR analysis of miR-193b-3p expression in HUVECs transfected with the miR-193b-3p mimic, mimic control, miR-193b-3p inhibitor, or inhibitor control. (C, D) miR-193b-3p binding to lnc_011797 (C) and WNK1 (D) was confirmed by dual luciferase reporter gene assay. (E) Lnc_011797 regulates miR-193b-3p expression. (F-I) RT-PCR (F, G) and western blotting (H, I) were used to measure WNK1 expression in cells transfected with the lnc_011797–specific lentivirus and the miR-193b-3p mimic or inhibitor. The data were normalized to the control group. Data are expressed as mean ± SD (*n* = 3). *P < 0.05, **P < 0.01, ***P < 0.001 (one-way analysis of variance followed by the least significant difference *post hoc* tests). HUVECs: Human umbilical vein endothelial cells; RT-PCR: real-time polymerase chain reaction; WNK1: with-no-lysine (K) 1.

Next, we sought to determine the mechanism by which lnc_011797 regulates WNK1. RNAhybrid and TargetScan database analysis revealed that miR-139b-3p binds to both lnc_011797 and WNK1 (**Figure 4A** and **Additional Table 4**). This led us to hypothesize that lnc_011797 regulates WNK1 expression by acting as a molecular sponge for miR-139b-3p. To test this, we transfected cells with miRNA mimic or an inhibitor, whose effectiveness was demonstrated by RT-PCR (**Figure 4B**). Furthermore, dual-luciferase assays showed that luciferase activity was inhibited in cells transfected with the miR-193b-3p mimic in combination with the lnc_011797 and WNK1 wild-type reporters (**Figure 4C** and **D**). However, in the lnc_011797 and WNK1 mutant reporter gene transfection groups, the miR-193b-3p mimic had relatively no effect on luciferase activity (**Figure 4C** and **D**). Taken together, these results suggest that lnc_011797 acts as a molecular sponge for miR-193b-3p, thereby regulating WNK1 expression.

**Additional Table 4:** Gene information

Gene	Chromosome	Length (bp)
Lnc_011797	Chr3	755
miR-193b-3p	Chr16	22

Next, we investigated whether lnc_011797 regulates miR-193b-3p expression. Lnc_011797 knockdown increased miR-193b-3p expression, while lnc_011797 overexpression inhibited miR-193b-3p expression (**Figure 4E**), indicating that lnc_011797 does in fact regulate miR-193b-3p expression. Subsequently, to show that miR-193b-3p participates in the regulatory relationship between lnc_011797 and WNK1, we assessed WNK1 expression at the mRNA and protein levels. To do this, we transfected cells with a lnc_011797-overexpressing lentivirus and found that cotransfection with miR-193b-3p mimic eliminated the upregulation of WNK1 induced by lnc_011797 overexpression (**Figure 4F** and **H**). Furthermore, cotransfection with the lnc_011797-silencing lentivirus and an miR-193b-3p inhibitor reversed the downregulation of WNK1 expression caused by lnc_011797 silencing (**Figure 4G** and **I**). Therefore, lnc_011797 interacts with miR-193b-3p to regulate WNK1 expression.

### Lnc_011797 promotes ferroptosis by inhibiting AMPK-mediated ACC phosphorylation via WNK1

**Figure 5:**
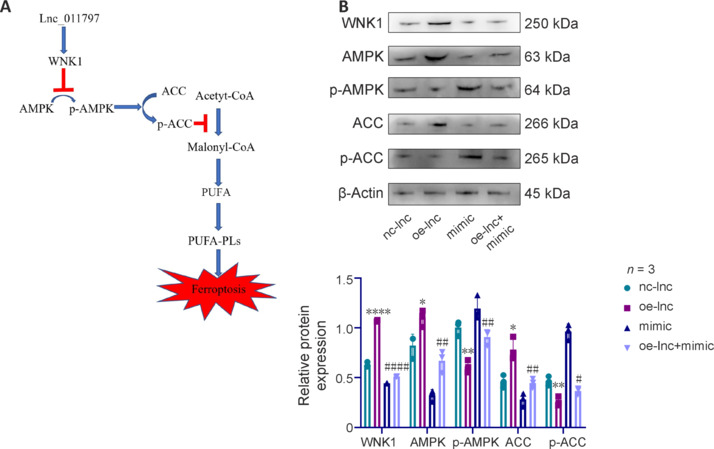
Ferroptosis is regulated by lnc_011797 via WNK1. (A) Mechanism by which lnc_011797 regulates ferroptosis. (B) Western blot analysis showed that p-AMPK and p-ACC were downregulated in lnc_011797–overexpressing cells. Data are expressed as mean ± SD (*n* = 3). *P < 0.05, **P < 0.01, ****P < 0.0001, *vs.* nc-lnc group; #*P* < 0.05, ##*P* < 0.01, ####*P* < 0.0001, *vs.* oe-lnc group (one-way analysis of variance followed by the least significant difference *post hoc* tests). ACC: Acetyl-CoA carboxylase; AMPK: AMP-activated protein kinase; PUFAs: polyunsaturated fatty acids; PUFA-PLs: polyunsaturated fatty acid-containing phospholipids; p-ACC: phosphor-ACC; p-AMPK: phosphor-AMPK; WNK1: with-no-lysine (K) 1.

We next asked how lnc_011797 affected the expression of ferroptosis-related proteins downstream of WNK1. No previous studies have linked WNK1 to ferroptosis, but studies have demonstrated that WNK1 inhibits phosphorylation of the T172 activation site on AMPK (Gallolu Kankanamalage et al., 2016). Moreover, ACC phosphorylation regulates ferroptosis (Lee et al., 2020). Therefore, we hypothesized that lnc_011797 may inhibit AMPK-mediated ACC phosphorylation through WNK1, thereby leading to ferroptosis. To test this, we assessed WNK1, AMPK, p-AMPK, ACC, and p-ACC expression levels by western blotting (**Figure 5**) and found that, in cells transfected with the lnc_011797-overexpressing lentivirus, WNK1, AMPK, and ACC expression levels were increased, while p-AMPK and p-ACC expression levels were decreased. Transfection with the miR-193b-3p mimic decreased WNK1, AMPK, and ACC expression and increased p-AMPK and p-ACC expression. These effects were reversed by cotransfection with the lnc_011797-overexpressing lentivirus and the miR-193b-3p mimic. These findings suggest that lnc_011797 regulate ferroptosis via the miR-193b-3p/WNK1 axis.

### Lnc_011797 knockdown inhibits ferroptosis and alleviates white matter lesions *in vivo*

**Figure 6:**
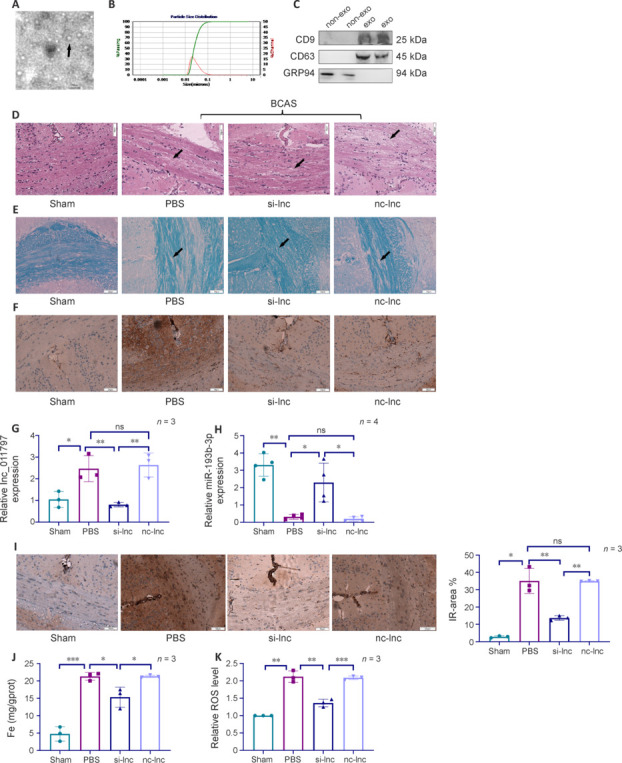
Lnc_011797 promotes WML formation in C57BL/6J mice. (A) TEM images showed that the exosomes (indicated by black arrows) were circular vesicles. Scale bar: 500 nm. (B) NTA results showed that the diameter of the enriched plasma exosomes was 30–100 nm. (C) Western blot analysis of the exosomal protein markers CD9 and CD63 and the negative control protein marker GRP94. (D) Pathological changes in the brain tissue were observed by HE staining. Nerve fiber disorders and vacuole-like changes in the brain tissue of the PBS and nc-lnc groups. In the si-lnc group, the lesions (indicated by black arrows) were clearly reduced. Scale bar: 50 μm. (E) Myelin in brain tissue was observed by LFB staining. The nerve fibers were broken, and the myelin sheath was sparse, in the brain tissue of mice in the PBS and nc-lnc groups. The degree of myelin sheath loss was reduced in the si-lnc group to levels similar to those seen in the sham group. The black arrows indicate lesions. Scale bar: 50 μm. (F) Glial cell activity was detected by immunohistochemical staining for GFAP (brown). Immunohistochemistry showed enhanced brown staining of the nerve fibers in the PBS group and nc-lnc group. Scale bar: 50 μm. (G) lnc_011797 expression in mouse brain tissue. (H) miR-193b-3p expression in mouse brain tissue. The data were normalized to the control group. (I) WNK1 immunoreactivity (brown) in the mouse brain was evaluated by immunohistochemistry. (J) Iron content in mouse brain tissue. (K) ROS levels in mouse brain tissue. Data are expressed as mean ± SD. *P < 0.05, ** *P* < 0.01, ***P < 0.001 (one-way analysis of variance followed by the least significant difference *post hoc* tests). GFAP: Glial fibrillary acidic protein; GRP94: glucose regulated protein 94; gprot: g protein; HE: hematoxylin and eosin; LFB: luxol-fast blue; NTA: nanoparticle tracking analysis; PBS: phosphate-buffered saline; ROS: reactive oxygen species; TEM: transmission electron microscopy; WML: white matter lesions; WNK1: with-no-lysine (K) 1.

Next, we sought to validate our *in vitro* data in a C57BL/6J mouse model of WMLs. First, exosomes were extracted from cells subjected to lnc_011797 knockdown or from negative control cells. Then, the exosomes were identified and evaluated by TEM, nanoparticle tracking analysis, and western blotting. TEM analysis of the exosomes revealed that they exhibited a circular shape and a size of approximately 100 nm (**Figure 6A**). Nanoparticle tracking analysis was used to analyze the size distribution of the exosomes in the range of 30–100 nm (**Figure 6B**). Western blot analysis revealed that the exosomes contained typical proteins (CD9 and CD63) but lacked cellular proteins (GRP94) (**Figure 6C**). Together, these findings indicate that we successfully isolated exosomes from cultured HUVECs (Théry et al., 2018). As shown in **Additional Table 5**, the neurobehavioral scores in the BCAS group were significantly lower than those in the sham group 1 month after surgery (*P* = 0.015). However, the neurobehavioral scores in the si-lnc group were higher than those in the BCAS group (**Additional Table 5**). HE and LFB staining demonstrated successful establishment of a mouse model of WMLs by inducing BCAS (**Figure 6D** and **E**). Exosomes were injected into the tail vein. To verify the effect of lnc_011797 knockdown on WMLs after BCAS, HE and LFB staining of brain tissue was performed. HE staining revealed disorganized nerve fibers and vacuole-like changes in the mouse brain tissue after BCAS. However, these pathological changes were considerably reduced in the si-lnc group compared with the PBS group and the nc-lnc group (**Figure 6D**). LFB staining revealed myelin loss, severe damage to the myelin sheath, nerve fiber disruption, and sparse myelin sheaths in mouse brain tissue after BCAS (**Figure 6E**). The degree of myelin loss in the si-lnc group was lower than that seen in the PBS and nc-lnc groups (**Figure 6E**). Next, to assess the effect of lnc_011797 deletion on the glial cell response to BCAS, we performed immunohistochemical analyses using an anti-GFAP antibody. BCAS clearly increased the area of the immune response, as indicated by GFAP staining. Moreover, the size of the GFAP-positive area decreased when lnc_011797 expression was inhibited (**Figure 6F**). Therefore, we successfully established a mouse model of WMLs and showed that lnc_011797 knockdown reduced histological damage to the white matter in this model.

**Additional Table 5:** Neurobehavioral score

Group	Neurobehavioral score
Sham	9±1
PBS	7.20±0.84*
si-lnc	8.80±0.84*
nc-lnc	7.4±1.14

Data are expressed as mean ± SD (*n* = 5). *P < 0.05, *vs*. sham group (one-way analysis of variance). PBS: Phosphate-buffered saline.

lnc_011797 expression in the brain was measured by RT-PCR. As shown in **Figure 6G**, lnc_011797 expression in the brain after BCAS was significantly increased (*P* = 0.03), consistent with our previous study’s results. lnc_011797 expression in the brain of mice decreased after injection of si-lnc exosomes into the tail vein (**Figure 6G**), demonstrating that the exosomes successfully penetrated the blood–brain barrier to affect lnc_011797 expression in the brain. In addition, miR-193b-3p expression was significantly downregulated in brain tissue after BCAS (*P* = 0.002; **Figure 6H**), and inhibiting lnc_011797 expression increased miR-193b-3p expression (**Figure 6H**). Next, we examined WNK1 levels in the brain. Immunohistochemical analysis showed that WNK1 expression increased after BCAS treatment (**Figure 6I**) and decreased in the si-lnc group compared with the PBS and nc-lnc groups (**Figure 6I**). Therefore, lnc_011797 affects WMLs through the miR-193b-3p/WNK1 signaling pathway.

To further demonstrate that lnc_011797 causes WMLs by promoting ferroptosis, we detected iron concentrations and ROS levels at WMLs sites. The levels of iron and ROS at WMLs sites after BCAS were greater than those in the sham group (**Figure 6J** and **K**). Furthermore, the concentrations of iron and ROS in the lncRNA_011797 knockdown group were markedly lower than those in the PBS and nc-lnc groups (**Figure 6J** and **K**).

Taken together, the results from our BCAS model indicate that lnc_011797 knockdown can reduce the development of WMLs in response to hypoperfusion. In addition, lnc_011797 promoted ferroptosis through miR-193b-3p/WNK1, leading to WMLs occurrence and development.

## Discussion

In our study, HUVECs were subjected to OGD to establish a cell model of WMLs (Choi et al., 2017). OGD significantly increased lnc_011797 expression and induced ferroptosis in the HUVECs. Furthermore, we found that lnc_011797 regulates ferroptosis and promotes WML formation through the miR-193b-3p/WNK1 signaling pathway. This mechanism was verified in a mouse model of WMLs.

The pathogenesis of WMLs is still not fully understood, and many studies suggest that WMLs are associated with chronic hypoperfusion. Studies have shown that endothelial function is significantly impaired in WMLs compared with normal controls, and the degree of endothelial function impairment is related to the degree of WMLs that are present (Rana et al., 2019; Inoue et al., 2023). LncRNAs are known to affect WMLs through a variety of pathways (He et al., 2017; Perry et al., 2018). Using high-throughput sequencing, we previously found that lnc_011797 is a biomarker of WMLs (Xu et al., 2023b). In the current study we found that lnc_011797 was significantly upregulated in our WMLs cell model. However, the mechanism by which lnc_011797 promotes WMLs development remained unclear. Previous studies have shown that ferroptosis plays a critical role in WMLs development and formation (Weiland et al., 2019; Jhelum et al., 2020). Shen et al. (2022) reported that inhibiting ferroptosis prevented oligodendrocyte death after intracerebral hemorrhage, thereby reducing white matter damage. A large number of recent studies have shown that lncRNAs can regulate ferroptosis in various ways (Li et al., 2023b; Xu et al., 2023a). For example, the lncRNA SNAI3-AS1 suppresses brain glioma progression by promoting ferroptosis *in vivo* and *in vitro* (Zheng et al., 2023). Abnormal iron homeostasis in brain tissue can induce brain cells to produce large amounts of ROS, ultimately causing catastrophic oxidative damage to sensitive subcellular structures (Stockwell, 2022). In the present study, OGD induced ultrastructural changes in mitochondria and significantly increased the iron and ROS contents of the cells, demonstrating that ferroptosis occurs in WMLs. Furthermore, lnc_011797 induces WMLs formation by promoting ferroptosis.

In the present study, a hypoxic cell model was established to simulate WMLs *in vitro*, and the results suggested that lnc_011797 promotes ferroptosis in WMLs. RNA FISH confirmed that lnc_011797 was located mainly in the cytoplasm. Therefore, lnc_011797 may be a ceRNA. The subcellular localization of lncRNAs affects many regulatory processes. In the nucleus, lncRNAs regulate transcriptional programs through chromatin interactions and remodeling (Melé and Rinn, 2016; Stockwell, 2022). In the cytoplasm, lncRNAs mediate signal transduction pathways, translation programs, and posttranscriptional control of gene expression (Yuan et al., 2017; Bridges et al., 2021). LncRNAs in the cytoplasm indirectly regulate target gene expression by acting as ceRNAs to inhibit miRNAs (Nojima and Proudfoot, 2022; Tan et al., 2023). Using a dual luciferase reporter analysis, we found that lnc_011797 acts as a ceRNA or sponge for miR-193b-3p, thereby mediating WNK1 expression. A previous study showed that miR-193b-3p is neuroprotective (Chen et al., 2020). Research has shown that miR-193b-3p may protect against focal cerebral ischemia-reperfusion injury (Lai et al., 2020). In our study, we found that miR-193b-3p can inhibit WNK1 expression, thus protecting against WMLs formation. This conclusion is consistent with previous studies of miR-193b-3p activity in the central nervous system. WNK kinases are an atypical family of protein kinases that regulate ion transport across cell membranes (Gallolu Kankanamalage et al., 2016). A previous study confirmed that WNK1 inhibits AMPK phosphorylation (Gallolu Kankanamalage et al., 2016). AMPK-mediated ACC phosphorylation leads to reduced lipid biosynthesis of polyunsaturated fatty acids (Lee et al., 2020). The higher the concentration of polyunsaturated fatty acids in cells, the greater the damage caused by lipid hydrogen peroxide and the greater the degree of ferroptosis (Bayır et al., 2020; Stockwell and Jiang, 2020). In our study, we found that lnc_011797 promoted ferroptosis in WMLs through WNK1. Furthermore, lnc_011797 overexpression promoted WNK1 expression, inhibited AMPK activity and ACC phosphorylation, and promoted ferroptosis, while a miR-193b-3p mimic reversed these changes. Thus, we conclude that lnc_011797 acts as a molecular sponge for miR-193b-3p, thereby regulating WNK1 expression and promoting ferroptosis. Our findings suggest that WNK1 may be a novel regulator of ferroptosis; however, the specific role of WNK1 in ferroptosis needs further validation in follow-up studies.

A previous study showed that mice develop WMLs and cognitive dysfunction after BCAS (Poh et al., 2021). Since lncRNAs are very unstable, exosomes were selected as carriers because of their small size, good biocompatibility, low toxicity, low immunogenicity, and ability to cross the blood–brain barrier (Zhang et al., 2022a). One study showed that exosomes carrying rifampicin can effectively treat central tuberculosis (Li et al., 2023a). Intravenously administered exosomes can repair white matter injury after intracerebral hemorrhage. In this study, exosomes containing lnc_011797-knockdown lentiviruses were injected into mice through the tail vein. The results suggest that these exosomes successfully crossed the blood–brain barrier successfully, alleviated myelin loss and glial activation, and inhibited white matter damage, which is consistent with our *in vitro* results. Neurobehavioral scores were used to further demonstrate neurobehavioral abnormalities in mice.

This study had several limitations. First, the role and mechanism of lnc_011797 in other WML-related cells remain unclear and should be investigated. Second, lncRNAs can regulate gene expression through various mechanisms, so other potential regulatory mechanisms of lnc_011797 in WMLs still need to be clarified. Moreover, our *in vitro* and *in vivo* findings should be confirmed in human studies. In the future we plan to determine the pharmacological effects of lnc_011797 on WMLs.

In conclusion, lnc_011797 promotes ferroptosis through the miR-193b-3p/WNK1 signaling pathway, leading to myelin loss in brain tissue and aggravating WMLs. The findings from this study help elucidate the regulatory mechanism of lncRNAs in the context of white matter injury and provide a new target and strategy for treating WMLs.

## Additional files:

***Additional Figure 1:*** *Flowchart of animal experiments.*

Additional Figure 1 Flow chart of animal experiments.
BCAS: Bilateral common carotid artery stenosis; PBS: phosphate-buffered saline.

***Additional Table 1:*** *Lentivirus sequence information.*

***Additional Table 2:*** *The PCR condition of miRNA.*

***Additional Table 3:*** *The PCR condition of lncRNA and mRNA*

***Additional Table 4:*** *Gene information.*

***Additional Table 5:*** *Neurobehavioral score.*

## Data Availability

*The data that support the findings of this study are available from the corresponding author upon reasonable request*.
