# Supplementary figures and images for: Lnc_011797 promotes ferroptosis and aggravates white matter lesions
Source: Neural Regen Res. 2024 Dec 16;21(5):2021–30. doi: 10.4103/NRR.NRR-D-24-00676 (PMC12694637; doi:10.4103/NRR.NRR-D-24-00676)

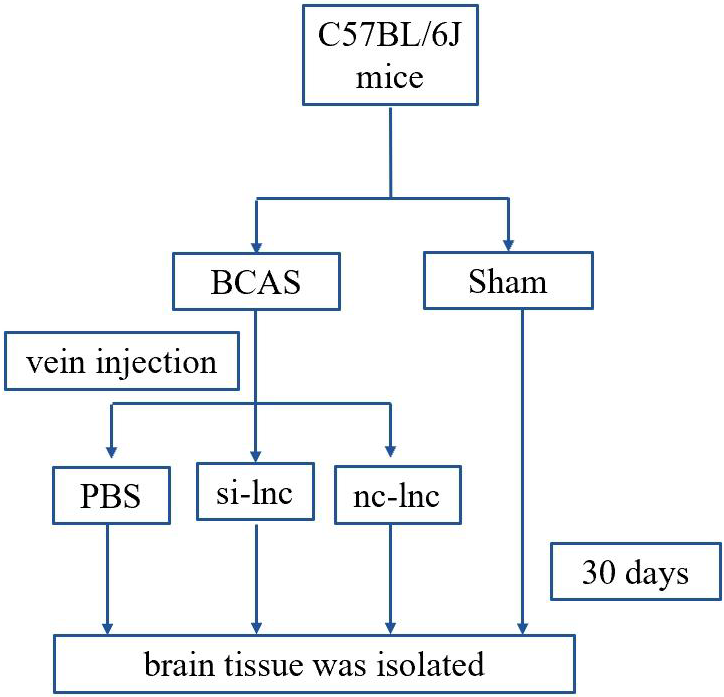

Supplement: Supplementary file 1 [file NRR-21-2021_Suppl1.tif]
